# SIT for African malaria vectors: Epilogue

**DOI:** 10.1186/1475-2875-8-S2-S10

**Published:** 2009-11-16

**Authors:** Harold Townson

**Affiliations:** 1 Liverpool School of Tropical Medicine, Pembroke Place, Liverpool, L3 5QA, UK

## Abstract

As a result of increased support and the diligent application of new and conventional anti-malaria tools, significant reductions in malaria transmission are being accomplished. Historical and current evolutionary responses of vectors and parasites to malaria interventions demonstrate that it is unwise to assume that a limited suite of tools will remain effective indefinitely, thus efforts to develop new interventions should continue. This collection of manuscripts surveys the prospects and technical challenges for applying a novel tool, the sterile insect technique (SIT), against mosquitoes that transmit malaria. The method has been very successful against many agricultural pest insects in area-wide programs, but demonstrations against malaria vectors have not been sufficient to determine its potential relative to current alternatives, much of which will hinge ultimately upon cost. These manuscripts provide an overview of current efforts to develop SIT and identify key research issues that remain.

## Epilogue

This supplement represents the collective efforts of many specialists in the field. As such, it provides an important resource for all who seek to understand the biology and behaviour of the *Anopheles gambiae *complex, and not solely for those with interests in the use of the sterile insect technique (SIT). The coverage is impressively broad, covering the problematic areas of reproductive number (how can we get reliable estimates of R_0_?), mating behaviour and male competitiveness, the maintenance of polymorphisms in laboratory-reared specimens, ingenious methods for sex separation, screening mosquitoes for release to reduce transmission of viruses and other potential mosquito pathogens, alternative methods to radiation sterilization for inducing sterility and the essential details of radiation biology of mosquitoes. The authors are to be congratulated on the thoroughness of their approach which, in part, reflects the knowledge and experience gained during the many years of use of SIT for pests of agricultural importance and the large scale and highly successful programmes for the eradication of the New World screwworm, *Cochliomyia hominivorax *[1].

SIT has helped eradicate the melon fly from Okinawa, stopped the invasion of southern Mexico by the Mediterranean fruit fly and helped eradicate the same species from Chile and southern Peru with enormous economic benefits for these countries, since their fruits can be imported elsewhere without quarantine. Bollworm moths and the codling moth have also been effectively controlled by employing SIT within an area-wide integrated programme [1].

However, SIT has not been without its sceptics and outright critics. During the screwworm eradication programme in Texas, it was argued that the decline of the screwworm population owed more to climatic factors than to the SIT programme, an interpretation that was roundly rejected [2]. Without the SIT programme, it is certainly possible that Africa would also have been blighted by the New World screwworm, following its accidental introduction into Libya. Despite political difficulties, US-reared sterile screwworms were employed successfully to eradicate this pest from Libya [3].

The successful eradication of the tsetse-fly *Glossina austeni *from Zanzibar also led to criticism of the idea of deploying SIT for tsetse control elsewhere in Africa [4], because of the costs and the plethora of potential vectors of trypanosomiasis. In a rejoinder, Hursey [5] argues that SIT is technically proven as a tool for tsetse eradication and feasible in practice. Given the low reproductive rate and population dynamics of tsetse populations, initial population suppression by a combination of tools, followed by sterile insect release could be feasible for some tsetse species or populations.

It is important to stress that in most control programmes employing SIT, the technique is part of an area-wide integrated pest management programme. In this respect, SIT fits well with the principle of Integrated Vector Management, a key strategic objective for WHO in vector control programmes [6,7]

In promoting SIT for the control of African malaria vectors, it will be important to define precisely the circumstances in which this technique may be deployed in a cost-effective manner. The programme in Sudan has the advantage that it addresses the perennial problem of preventing malaria vectors from becoming re-established in Egypt, as well as having the potential to eradicate *Anopheles arabiensis *from its marginal areas in the arid regions of Sudan [8,9]. Similarly, in La Réunion, where this species was accidentally introduced in the mid 19th Century, there are good opportunities for a programme of eradication based on the integration of conventional control measures with SIT.

It is worth examining why research support and donor attention should be considered for the sterile insect technique (SIT) for control of malaria vectors in Africa, particularly at a time when advances in malaria control have helped reduce the burden of disease dramatically in several African countries.

Artemisinin-based combination therapy (ACT) has brought new and effective methods for treating malaria that allow for shorter treatment courses and being in combination, help delay the onset of drug resistance [10]. In addition, by reducing malaria infection in the population, the use of ACT can bring about a reduction in transmission [11].

In Africa, malaria vector control currently relies primarily on the use of mosquito nets treated with pyrethroid insecticides (insecticide-treated nets or ITNs) and the indoor spraying of houses with residual insecticides (indoor residual spraying or IRS), including DDT. In Rwanda, external funding including the US President's Malaria Initiative (PMI), has backed four key interventions [12]. These are the use of ITNs, IRS, intermittent preventive treatment for pregnant women (IPTp) and the diagnosis of malaria and treatment with ACT. Following their deployment, there have been dramatic reductions in malaria prevalence (to under three percent) and an overall reduction in under-five mortality of 32 percent between 2005 and 2008. On Zanzibar, a similar strategic approach has led to a reduction in malaria positive blood smears in children under the age of two from 22 percent to less than 1 percent [13]. These are significant achievements within 3-4 years of programme implementation. Similar programmes are being rolled out in Angola, Benin, Ethiopia, Ghana, Kenya, Liberia, Madagascar, Malawi, Mali, Mozambique, mainland Tanzania, Uganda and Zambia.

So why look to new technologies? A key reason is that the evolution of drug resistance to anti-malarials and of insecticide resistance in malaria vectors has the potential to reverse the important benefits so far achieved. The spread of high-level pyrimethamine resistance in Africa threatens to curtail the therapeutic lifetime of antifolate anti-malarials in the continent [14]. There is growing evidence of artemisinin resistance in malaria parasites around the Thai-Cambodian border [10]. Resistance has arisen partly due to the widespread use of artemisinin monotherapy and may have been accentuated by the use of sub-optimal doses or counterfeit versions of the drug. The evidence of artemisinin resistance has led to urgent plans for containment by switching from artemisinin monotherapy to ACT, a plan that can only succeed if all malaria were eliminated from western Cambodia, since the last few infections to be cleared will almost certainly be the most resistant [10]. The development of artemisinin resistance in the Myanmar-Thai-Cambodia region is not without risks for Africa. There is strong evidence that chloroquine resistance in the African continent was due to the importation from South-East Asia of a mutant allele [15]. If this also happened with artemisinin resistance, the consequences could be grave.

Similarly, resistance to pyrethroid insecticides in malaria vectors is increasingly reported from Africa, some of which may result from the common use of pyrethroids in agriculture [16]. Santolamazza *et al *[17] have described the widespread distribution of the *kdr *resistance alleles in African populations of the M and S-forms of *An. gambiae. *In West Africa (Senegal to Nigeria), the L1014F allele was present at frequencies of over 50% in most sites. In the region from Cameroon to northern Angola and eastwards to Uganda, both *kdr *resistance mutations were present in most sites. It is unclear whether the level of resistance attained is sufficient to reduce the efficacy of ITNs that are fully loaded with insecticide, but the history of insecticide resistance in anophelines suggests that in time, resistance could compromise the use of ITNs. In Uganda, adult *An. gambiae s.l. *raised from wild-caught larvae showed survival rates as high as 85% with a WHO discriminating concentration of DDT and there was also significant resistance to permethrin (38.5% survival) and deltamethrin [18].

In the light of these findings, it cannot be assumed that pyrethroid-impregnated ITNs and IRS will continue to be as effective in the future as in the recent past. Additional tools will be needed to bring about effective control of African malaria vectors.

One aspect of malaria vector control that has received insufficient attention is the application of the principles of integrated vector management (IVM) briefly discussed earlier. This concept envisages a multi-sectoral approach to control, and accepts a greater need for community involvement, whether it be through better management of surface water breeding sites, the siting of housing, or improvements leading to more mosquito-proof housing. Programmes following IVM principles are being rolled out in several WHO regions, including AFRO and EMRO, and these can be expected to lead to improvements in approaches to malaria vector control. Chanda *et al *[19] have described the application of these principles in the development of the Zambian Malaria Control Programme with the Roll Back Malaria (RBM) partners. Given the history of SIT as part of an area-wide integrated progamme, the SIT programme described here can be considered as yet another tool within IVM.

One conclusion from the above analysis is that all possible methods of malaria vector control need to be explored if success in the long-term aim of ridding Africa of malaria is to be achieved. The series of articles in this supplement fully explore the various components required for an effective SIT programme, but it is worthwhile to consider what may be the remaining obstacles to successful SIT for *An. arabiensis*.

In their article, Howell and Knols consider the level of knowledge of male mating behaviour in anophelines. They rightly identify areas of knowledge that need to be strengthened, and point out that in some cases colonization of mosquitoes is associated with assortative mating behaviour. The evidence available suggests that this may not be a problem with SIT for *An. arabiensis *since colonies from La Réunion and continental Africa appear to have mated freely [20]. Nevertheless, a better understanding of male swarming sites may be required if male release is to have an optimal impact on the resident female mosquito population. Although aspects of sexual recognition in mosquitoes have been understood for some time, it is only in the recent past that the auditory behaviour of female mosquitoes has been examined in any detail. Recent studies have demonstrated an unexpected synchronization of wing-beat frequencies in male/female pairs of diverse genera of mosquitoes [21-23]. Colonization can affect male and female size and wing beat frequencies, hence it could be important to ensure that released males do not differ in size from natural males. This most useful chapter by Howell and Knols [24] ends with a series of important research questions, the answers to which may impact on the success of an SIT programme.

One aspect not considered in this supplement is a cost-benefit analysis of SIT. Carrying out cost-benefit analysis of control programmes is fraught with difficulty, particularly if scale-up is not given appropriate consideration, as the arguments over tsetse eradication proved [5]. However, if SIT is to make a significant impact in the control of African malaria vectors, particularly outside of the Sudan trial area, progress must be made in determining the overall costs and how these compare with more conventional methods deploying insecticides or anti-malarial drugs. Mumford [25] has provided a useful framework for cost benefit analysis of SIT programmes; a similar approach will be essential if other major donors are to be persuaded to help fund SIT programmes.

In conclusion, it would be wrong to view SIT as a panacea for the problems of malaria vector control and this is clearly not the objective of this IAEA-supported programme. However, given the current heavy reliance on insecticides and the risks this entails, all those involved in malaria vector control need to consider a more integrated approach, meeting the principles of IVM.

A great deal will be learned about the feasibility of SIT from this programme, once male mosquito releases start in earnest. Given the continuing support of IAEA, our understanding of the population dynamics and behaviour of *An. arabiensis *will be greatly extended with consequent benefits not just for SIT programmes, but for all those concerned with malaria vector control in Africa.

## Competing interests

The author declares that they have no competing interests.
